# A crop cultivation monitoring platform for evaluating the early growth of cucumbers

**DOI:** 10.3389/fpls.2026.1869455

**Published:** 2026-09-11

**Authors:** Deyi Lei, Zaibiao Zhu, Penghong Shen, Zhibo Zhong, Mohamed Ahmed Moustafa, Jieyu Xian, Hongbin Wu, Xiuqing Fu

**Affiliations:** 1 College of Engineering, Nanjing Agricultural University, Nanjing, China; 2 College of Horticulture, Nanjing Agricultural University, Nanjing, China; 3 College of Smart Agriculture, Nanjing Agricultural University, Nanjing, China; 4 Institute of Farmland Irrigation and Water Conservancy & Soil and Fertilizer, Xinjiang Academy of Agricultural Reclamation Sciences, Shihezi, China; 5 Faculty of Agricultural Engineering, Al-Azhar University, Cairo, Egypt

**Keywords:** cotyledon area, crop cultivation monitoring platform, germination rate, YOLOv8 models, ZnO-NPs suspension induction

## Abstract

The precise temporal characterization of early growth dynamics in crops under abiotic stress is critical for stress resistance management in smart agriculture. However, traditional manual measurement methods struggle to achieve high-throughput and accurate quantification of multiple phenotypic parameters over continuous time series. To address this, we developed a full-time-series crop growth monitoring system that continuously collects fine-grained image data of cucumber seed germination and seedling growth under soil culture conditions, constructing an annotated dataset for identifying germination status and quantifying cotyledon area. The YOLOv8n-F_SGX and YOLOv8-seg models were developed and deployed, achieving detection and segmentation accuracies of 0.978 and 0.987, respectively, which enabled automatic monitoring of germination rate and precise extraction of cotyledon area. To further investigate the mitigating effects of nanomaterial priming on salt stress, we conducted germination and seedling growth experiments on cucumber seeds using a series of ZnO-NPs (particle size 30 nm) suspension concentrations at different salt stress levels. The results demonstrated that under salt stress conditions of 0–150 mmol·L^−1^, priming with 100 mg·L^−1^ ZnO-NPs resulted in optimal germination dynamics (highest germination rate and fastest germination speed), along with the largest cotyledon area and highest growth rate in seedlings. In contrast, when the ZnO-NPs concentration increased to 400 mg·L^−1^ or higher, it significantly inhibited seed germination and seedling growth.

## 1 Introduction

Approximately 7% of the global land is currently threatened by salinity, with the area of saline–alkali land continuously expanding, which hinders the sustainable development of agriculture (Zhao et al., 2020). Seeds are the first link in agricultural production, directly affecting crop yield and quality (Zhang et al., 2021). Currently, breeding crop varieties that can adapt to saline–alkali environments has become a hot research topic for agronomists, often evaluating salt tolerance during the most sensitive stages of seed germination and seedling growth in crops (Ibrahim, 2016).

Cucumber is a widely beloved and popular vegetable, with a global cultivation area reaching 233 hectares (Zhang et al., 2020). In recent years, greenhouse agriculture technologies have rapidly developed in saline–alkali areas such as Xinjiang in China. Enhancing the early growth vigor of cucumbers in saline soils, improving their yield and quality, and meeting the demand for vegetables in salinity-affected areas have remarkable practical implications. Using nanoparticles (NPs) in seed priming treatments can alter seed metabolism and signaling pathways, thereby exerting sustained positive effects on crop growth and development in saline–alkali environments (Landa, 2021). ZnO-NPs can act as auxiliary factors for metabolism and regulatory enzymes, alleviating the negative effects of salt stress on seed germination and seedling growth processes in plants (Alhammad et al., 2023). Abdel Latef et al. (2017) used ZnO-NPs suspension to prime Lupinus termis seeds, alleviating the negative impact of 150 mmol·L^−1^ NaCl soil on the seedlings. Wang et al. (2020) found that wheat seeds treated with ZnO-NPs suspension can enhance photosynthetic electron transfer and sucrose biosynthesis efficiency in leaves under salt stress, thereby improving wheat’s salt tolerance. Ouyang et al. (2024) studied the effects of ZnO-NPs suspension priming and salt stress on radish seed germination and found that low concentrations of ZnO-NPs priming can increase the germination rate and germination energy of radish seeds under salt stress.

In summary, appropriate concentrations of ZnO-NPs suspension priming have a certain promoting effect on seed germination and seedling growth in saline–alkali environments. Therefore, further evaluation of the aforementioned promoting effects is necessary. The impact of stress environments on seed germination and seedling growth can be quantified by calculating indicators such as seed germination rate and seedling leaf area. However, the traditional manual measurements are difficult to obtain continuous indicators such as germination rate and leaf area. In recent years, image processing technology and machine learning algorithms, such as crop classification, feature recognition, and trait quantification, have been widely applied in the processing and analysis of crop phenotype data (Kamilaris and Prenafeta-Boldú, 2018; Singh et al., 2021; Murphy et al., 2024). In the field of seed germination and seedling growth, Li et al. (2024) proposed the RT-DETR-SoilCuc object detection model, achieving accurate identification of cucumber embryonic roots in soil cultivation backgrounds. Wu et al. (2024) constructed the YOLOv8-segANDcal model for soybean embryonic root feature segmentation and length calculation, capturing the morphological evolution of embryonic root contours with germination time. Colmer et al. (2020)developed the cost-effective phenotype platform SeedGerm for automatic seed imaging and machine learning-based crop seed germination phenotype analysis. Awty-Carroll et al. (2018) implemented the recognition and classification of foxtail seed germination phenotypes by using the k-NN machine learning algorithm. de Medeiros et al. (2020) proposed a model on the basis of interactive and traditional machine learning methods to classify soybean seeds and seedlings based on their appearance and physiological potential. Overall, the breeding industry’s perspective has shifted towards data-driven approaches due to the integration of big data analysis and phenotype platforms (Yan and Wang, 2023).

Therefore, a crop cultivation monitoring platform was developed to accurately quantify the germination rate of cucumber seeds and the leaf area of seedlings over continuous time and evaluate the effects of ZnO-NPs suspension priming on the early growth of cucumber under salt stress. Image data of cucumber seed germination and seedling growth under soil cultivation conditions were collected. A labeled dataset was constructed for identifying seed germination and quantifying leaf growth. The characteristics of cucumber seedling and seedling growth were recognized and segmented. Vitality indicators, such as seed germination rate, germination energy, germination index, and seedling leaf area, during the seedling and seedling stages were obtained. Composite experiments on cucumber seed germination were conducted under soil cultivation conditions. Changes in the vitality indicators of cucumber seed germination and seedling growth under different concentrations of salt stress induced by ZnO-NPs suspension priming were analyzed. This paper presents a combined experiment on cucumber seed germination and cotyledon growth conducted under soil-based cultivation conditions. Unlike the aforementioned studies, which focused solely on a specific growth stage of crops under hydroponic conditions, this study utilizes soil-based cultivation to more accurately simulate the growth conditions of cucumbers in the field. Furthermore, our study is not limited to the germination stage of cucumber seeds but extends through to the cotyledon growth stage. By continuously monitoring various vitality indicators during these two critical growth stages, we can more comprehensively investigate the effects of ZnO-NPs on the salt tolerance of cucumbers during their early growth stages. This work provides meaningful references for exploring the impact of ZnO-NPs suspension priming on the salt tolerance of cucumber during the early growth stage.

## 2 Materials and methods

### 2.1 Development of crop cultivation monitoring platform

**Figure 1:**
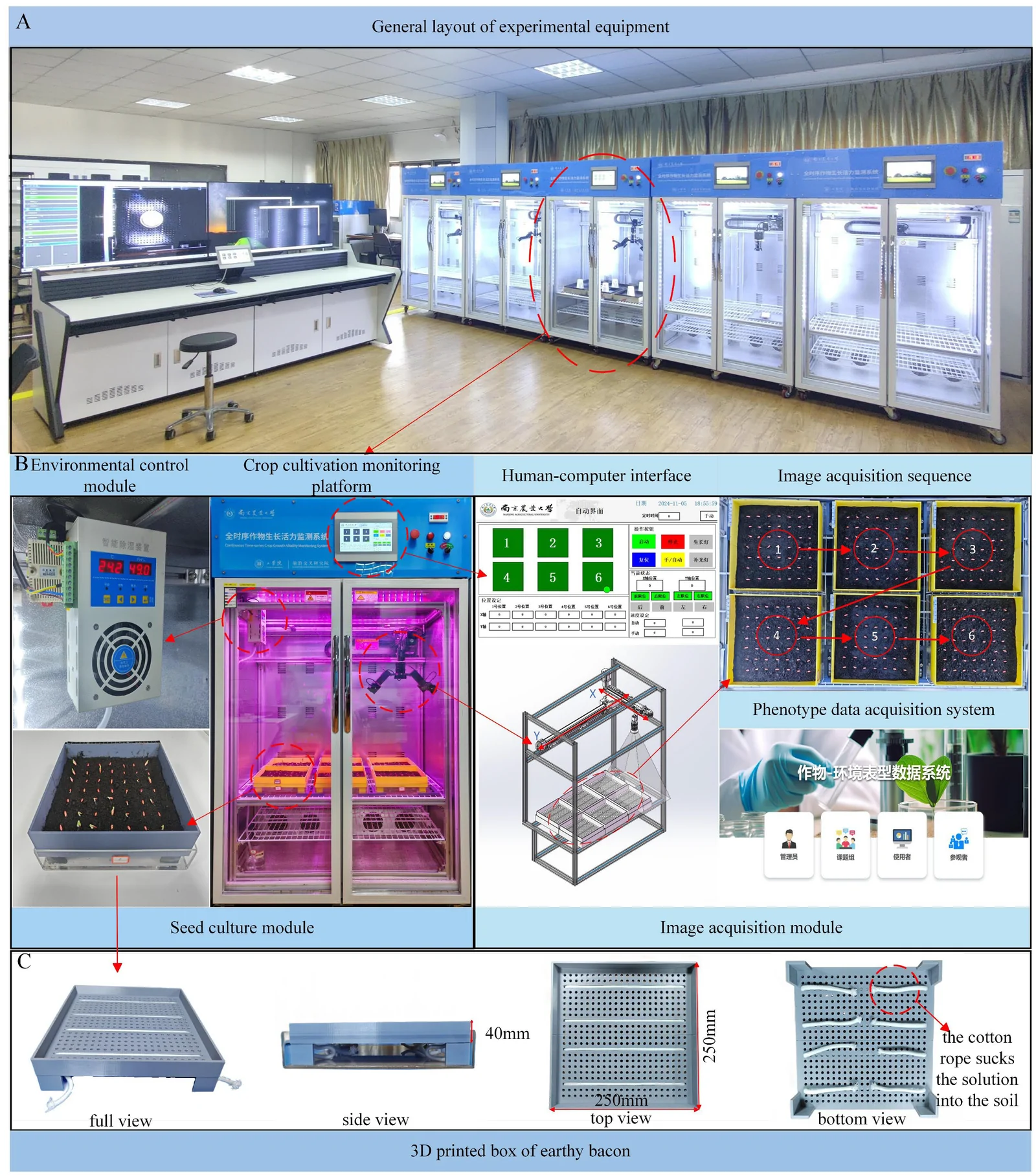
Crop cultivation monitoring platform: **(A)** overall layout of the system. **(B)** system components. **(C)** soil-rooting box.

The crop cultivation monitoring platform is shown in **Figure 1**. It mainly consists of an environmental control module, a seed germination cultivation module, a track-type high-throughput image acquisition module, an image processing module, and a human-machine interaction interface module.

The crop cultivation monitoring platform integrates three modules in accordance with the requirements of standard germination experiments: seed germination cultivation, environmental control, and image acquisition. It can simulate different environmental conditions required for seed germination experiments indoors and automatically and continuously acquire full-time sequence growth images of seeds from the budding stage to the seedling stage.

The environmental control module has an automatic heating function to achieve a constant temperature condition inside the box, and it controls the size of the ventilation openings to ensure that the seeds are in a suitable humidity environment. The system can also automatically control the on/off of the LED supplementary light at regular intervals, providing sufficient illumination for the camera to capture images. A temperature and humidity sensor is installed inside the box to enable monitoring of the internal environmental status in real time.

The rail-type image acquisition module controls the servo motor through a PLC program to achieve high-precision movement of the guide rail in the X-axis and Y-axis directions, enabling the RGB imaging sensor installed on it to automatically acquire full-time sequence germination and growth images of cucumber seeds in six areas inside the box. These images are then transmitted to a PC for storage through a GigE Gigabit network high-speed interface. The human-computer interaction interface module allows for manual setting of environmental parameters, such as temperature and illumination, and the interval time and area of image acquisition.

### 2.2 Image acquisition and dataset construction

A total of 588 uniformly textured and plump Zhilv 0116 cucumber seeds (Nanjing Green Collar Seed Industry Co., Ltd.) were selected for soil cultivation experiment to obtain full-time sequence germination and growth images of cucumber seeds from the budding stage to the seedling stage. In accordance with the soil conditions required for cucumber seed germination, the experimental soil (Tibet Wenbensheng Soil Management Co., Ltd.) is a natural sandy soil that has undergone dual impurity removal and sterilization by strong ultraviolet rays and microwaves. The pH value is between 5.5 and 7.6, and the soil is loose, breathable, not prone to hardening, and pure and harmless.

**Figure 2:**
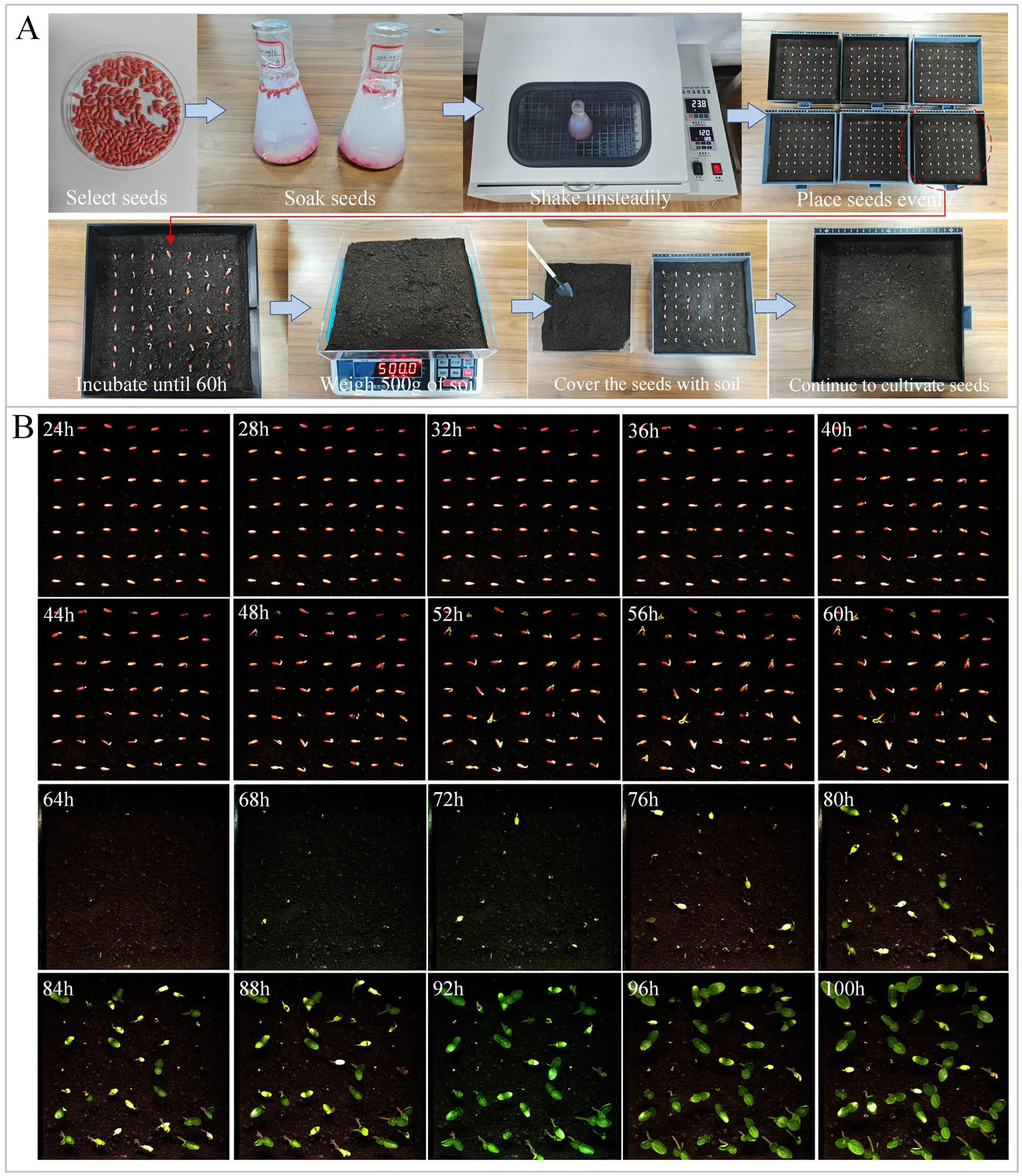
Image acquisition during budding and seedling stages: **(A)** equipment parameters and cultivation conditions. **(B)** experimental procedure.

The selected cucumber seeds were soaked in deionized water for 6 h and then dried. Next, 500 g of sandy soil (with a thickness of approximately 15 mm) was weighed using a high-precision electronic weighing scale and spread evenly. The seeds were evenly arranged on the soil layer in a 7 × 7 arrangement for germination. Then, the soil-rooting box was placed in an ambient temperature of 25 °C ± 1 °C for dark cultivation. The more specific experimental parameters are shown in **Figure 2A**. After 90% of the seeds showed white tips (60 h), 350 g of sandy soil (with a thickness of approximately 10 mm) was weighed, and the germinated cucumber seeds were evenly covered. Germination continued under the condition of 14 h of light and 10 h of darkness. Images were acquired at an interval of 20 min from the budding stage to the seedling stage for 7 days. The specific experimental process is shown in **Figure 2B**. We organized the numerous images collected during the preliminary experiment into time series and by experimental group. To ensure that the training dataset remained of a reasonable size while covering all stages of cucumber seed germination and cotyledon growth, we randomly selected 600 images from the dataset. Additionally, to prevent data leakage before constructing the dataset, we replaced the images in the test set with those obtained during the formal experiment, rather than using images from the preliminary experiment that were used for training, to avoid overstating the reported accuracy.

The germination and growth images of 12 seed trays in two boxes were cyclically acquired for 5 days through the process shown in **Table 1**. The experiment was divided into two stages, with the soil covering operation as the time interval point between the two stages. The first 60 h were the budding stage, and the last 60 h were the seedling stage. During this period, a total of 2160 budding stage images were acquired. Considering that the germination phenomenon of cucumber seeds was not obvious within the first 30 h and they started to germinate from the 30th h, a total of 1080 germination images acquired within the first 30 h were excluded. In addition, blurry photos caused by external factors, such as camera shaking, network fluctuations, and exposure failures, were further excluded. The remaining 1000 high-quality images were used to manually construct the dataset for the budding stage model.

**Table 1:** Key parameters for preliminary soil-based cucumber cultivation trials.

Experimental conditions	Parameters
Incubation temperature	25 ± 1°C
Incubation humidity	70%~90%
Duration of cultivation	144h
Acquisition interval	20min
The number of seeds per plate	49
Camera model	MV-CA050-20GC
Camera pixels	5 million
Image format	JPG
Image resolution	2592×2048

Image classification is one of the core tasks in computer vision, aiming to assign predefined class labels to images (Jiang and Li, 2020). LabelImg software was used to label the sorted images. The label types were divided into “seed” and “root,” where the number of “seed” labels represents the number of seeds, and the number of “root” labels represents the number of germinated seeds. The emergence of the radicle of the seed through the seed coat is regarded as germination. Python code was used to batch convert the XML files automatically generated by LabelImg software into TXT coordinate label files to improve the efficiency of data processing and match the algorithm design.

**Figure 3:**
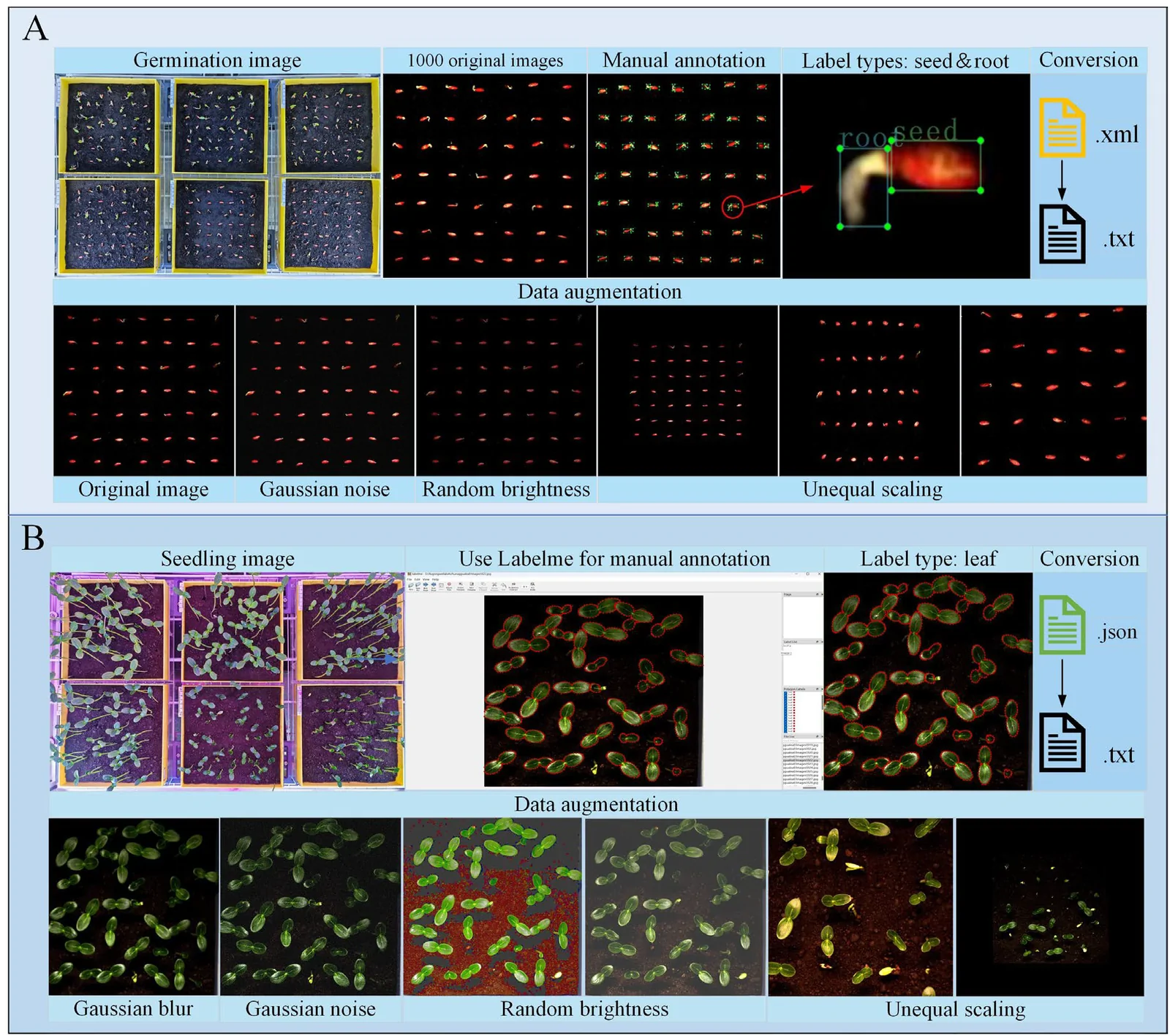
Production process of the dataset: **(A)** production flow of budding stage dataset. **(B)** production flow of seedling stage dataset.

Data augmentation aims to artificially increase the size, quality, and diversity of data to ensure good model performance (Badgujar et al., 2023; Mumuni and Mumuni, 2022). A total of 100 images from the above original images and corresponding label files were randomly selected for data augmentation to simulate various situations that may be encountered in the real world, such as different lighting conditions, angles, and occlusions. By adding Gaussian noise, non-uniform scaling, and randomly adjusting the brightness (Chen et al., 2023), variants of the original data were created to increase the size and diversity of the dataset, obtaining 300 data-augmented images and label files. Finally, the resulting dataset contained 1300 images and approximately 63,700 seed labels, which were divided into training, validation, and test sets in the ratio of 8:1:1. The operation process is shown in **Figure 3A**.

After the soil covering operation was completed, the soil-rooting box was continued to be placed in the incubator for constant temperature cultivation, and the full-time sequence image acquisition for the seedling stage was started. The seedling stage growth experiment lasted for 60 h, and 2160 full-time sequence growth images of seedlings in six workstations were acquired. Given that the seedlings did not break through the soil layer in the early stage and the cotyledons of some seedlings were seriously occluded in the later stage of growth, after removing these types of images, the 600 selected high-quality images were used to construct the seedling stage dataset.

Instance segmentation aims to assign each pixel in an image to a specific class label (Rawat et al., 2022), thereby distinguishing different objects in the image and their boundaries. Therefore, the accuracy requirement for segmentation reaches the pixel level (Mo et al., 2022). The segmentation results need to accurately distinguish each object in the image, including the details of edges and corners. For this purpose, the “Polygons” (polygon) tool in Labelme software was used to perform fine manual multipoint closed labeling on each leaf contour in the image. The effect is shown in **Figure 3B**. The labeled leaf area was named “leaf.” Given that the label files saved by the software were in.json format, a program script was used to uniformly convert the label files into.txt text format to facilitate the parsing of labeling information. Data augmentation was performed on the basis of 600 original images to improve the robustness of the algorithm so that it can handle images under different lighting conditions, blurriness, background complexity, and different viewing angles. By using data augmentation methods, such as random brightness, Gaussian blur, and non-uniform scaling and adding Gaussian noise to simulate the image effects in the complex real environment, new variants were generated from the existing dataset to increase the diversity of the data. Specifically, the 1300 images for the budding stage were divided into training, validation, and test sets at a ratio of 8:1:1. To prevent data leakage, the 130 test images were exclusively selected from the formal experiment, whereas the training and validation images were from the preliminary experiment, ensuring no overlap between training and test sets. The compositions of the two datasets are shown in **Table 2**.

**Table 2:** Composition of the cucumber sprout growth stage dataset.

Dataset composition	Budding dataset	Seedling dataset
Original image	1000	600
After data augmentation	1300	1200
Proportion of divisions	8∶1∶1	8∶1∶1
Train	1040	960
Test	130	120
Val	130	120

### 2.3 Selection of germination image detection models and evaluation metrics

The single-stage detector YOLO has rapidly gained popularity in agriculture owing to its real-time detection capabilities, good accuracy, and compatibility with resource-constrained devices (Badgujar et al., 2024). On the basis of the previous versions of YOLO, YOLOv8 improves the accuracy of object detection by introducing new architectures and optimization techniques. In seed germination images, the YOLOv8 model can effectively distinguish among seeds, soil, and other background elements and identify the germination characteristics at different stages (Yi et al., 2023; Hussain, 2023). YOLOv8n is the smallest variant and can offer the fastest speed (Bai et al., 2023). Considering the requirements for model parameters in edge device deployment, YOLOv8n was chosen as the initial detection model, and the abovementioned 1300-image dataset was used for model training.

On the basis of Windows 11 operating system, a virtual environment was built to train object detection models with the help of the high-performance computing service platform OpenBayes. The framework for deep learning is based on the Ultralytics software package and PyTorch 2.0.1. The main settings of the model training parameters are shown in **Table 3**.

**Table 3:** Main hyperparameters for training the object detection model.

Index	Parameters
GPU	NVIDIA GeForce RTX 4090
Epochs	100
Batch Size	16
Image Size	640×640
Learning Rate	0.01
Momentum	0.937
Weight Decay	0.0005

Selecting appropriate evaluation metrics is crucial for comparing and improving the performance of object detection algorithms. The evaluation metrics of object detection algorithms are mainly used to measure the performance of the model in detection tasks, including aspects such as accuracy, speed, and robustness. Precision (P), recall rate (R), average precision (AP), and their mean value (mAP) were taken as the indicators for evaluating the detection accuracy to accurately evaluate the recognition effect of the object detection model on cucumber seeds and radicles as well as the training speed. Moreover, the number of parameters (Params) and the number of floating-point operations (FLOPs) were taken as the indicators for evaluating the complexity of the germination image detection model. Params and FLOPs are two important indicators for measuring the complexity of the model; they represent the number of parameters and the amount of computation of the model, respectively. The smaller they are, the more efficient the model is, which means it can run under lower computing resources and requires less training time.

### 2.4 Evaluation tests of the YOLOv8n germination image detection model

**Figure 4:**
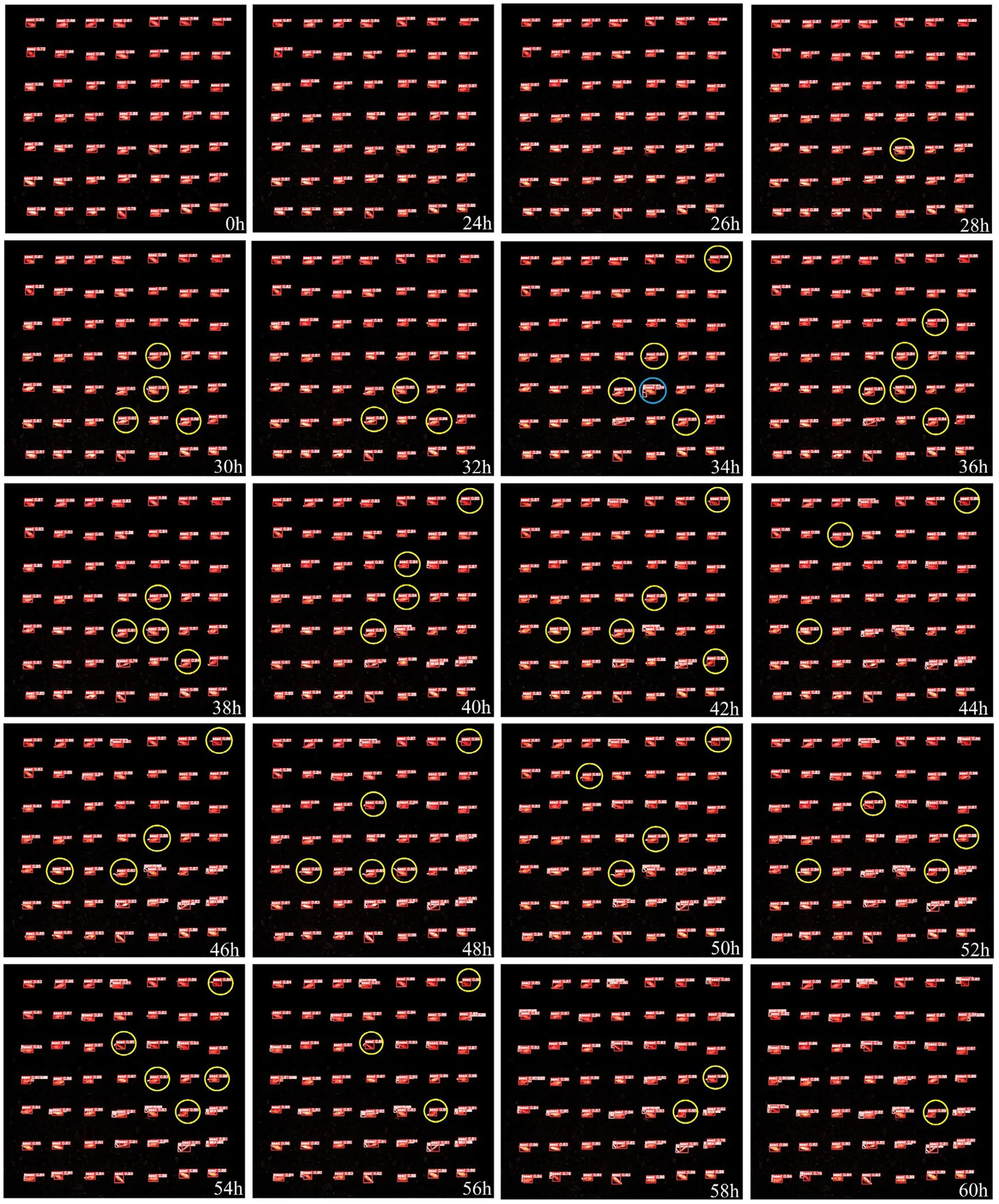
Detection effect diagram of the YOLOv8n model.

After the above training was completed, one image was selected every 2 h within the 24–60 h of the budding stage for prediction to evaluate the detection effect of the model on cucumber seed germination images. The prediction results are shown in **Figure 4**, where the blue circles represent false detections, and the yellow circles represent missed detections. The germination phenomenon of seeds was not obvious within 0–26 h. At this time, the model showed a good recognition effect on “seed,” with almost no missed or false detections, and could maintain a relatively high confidence level (between 0.8 and 0.9). The seeds started to germinate at the 26th h. Due to the small size of the radicle in the early stage, the model had certain defects in detecting small targets. At this time, the phenomenon of missed detections began to occur, and as the number of germinated seeds increased, the number of missed detections gradually increased. More missed detections were found between 30 and 52 h. By the 34th h, even the phenomenon of false detections occurred. After the 56th h, when the radicle developed to a certain length, the number of missed detections decreased significantly.

The above prediction results showed that the YOLOv8n object detection model can achieve the goal of identifying cucumber germination in the soil cultivation environment. However, the demand for the ability to identify and detect small targets remains high due to the small size of the radicle in the early stage of seed germination. Moreover, the time and computing resources required for 100 rounds of training are huge. How to maintain relatively small Params and FLOPs while improving the detection accuracy of the model for small targets, such as radicles, is one of the goals for improvement. The parameters of the initial YOLOv8n model are shown in **Table 4**.

**Table 4:** Parameters of the initial YOLOv8n model.

Model parameter	Metric
P	0.934
R	0.911
mAP50	0.947
Params	3M
FLOPs	8.1G

### 2.5 Improvement schemes for the YOLOv8n germination image detection model

**Figure 5:**
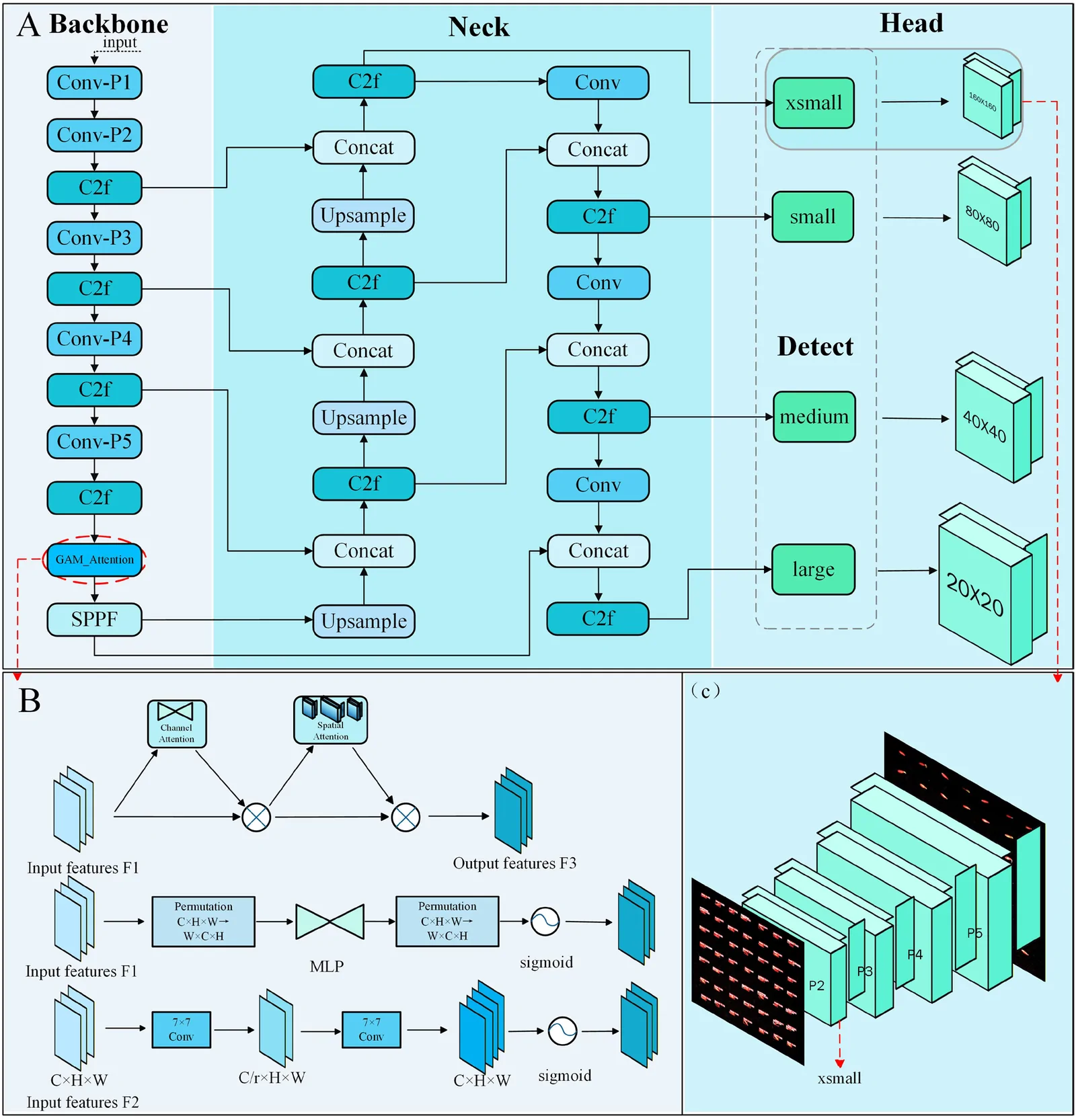
Network structure diagram of the improved model: **(A)** overall network structure. **(B)** principle of GAM attention mechanism. **(C)** small target detection layer.

**Figure 5A** shows the overall network structure of the improved model. The following improvements were made on the basis of the original model to further enhance the model’s ability to detect and recognize small targets, such as radicles, while avoiding a significant increase in the model’s Params and FLOPs and reducing the deployment difficulty and training time of the model:

1. Adding the Focal_SIoU loss function in addition to the existing loss functions such as CIoU: The Focal_SIoU loss function is an improved loss function for object detection models that combines Focal Loss and Scaled Intersection over Union (SIoU). These loss functions can alleviate the imbalance problem between positive and negative samples, increase the model’s attention to small targets, and enable the model to have a good adaptability to the phenomenon that the number of seeds far exceeds the number of radicles in some germination images.
2. Adding the GAM (Global Attention Mechanism) to the backbone network: The attention mechanism can improve the detection accuracy of small targets (Li et al., 2023), and its principle is shown in **Figure 5B**. The core idea of GAM is to enable the model to focus on the most important parts of the input data through the global attention mechanism, thereby enhancing the model’s ability to capture key information. The GAM helps focus on the continuously changing size of the radicles during the germination process of cucumber seeds, thus strengthening the model’s ability to capture radicles.
3. Adding a small target detection layer: The evaluation experiments of the original model demonstrated that the model has insufficient ability to capture small radicles in the early stage of seed germination. The presence of this flaw may be because the standard YOLO only includes three detection output layers (P3–P5), which reduce the image dimension with sampling rates of 32, 16, and 8 to detect large, medium, and small objects (Bochkovskiy et al., 2020). Among them, the largest detectable feature map is 80 × 80, and only targets with a size of 8 × 8 or larger can be detected. A fourth output layer was added on the original basis, as shown in **Figure 5C**, newly adding a detection feature map of 160 × 160 for detecting targets with a size of 4 × 4 or larger, to improve the model’s ability to detect radicles in the early stage of germination.

### 2.6 Ablation experiments of the cucumber seed germination detection model YOLOv8n-F_SGX

The models were trained using the same batch of datasets to verify the effectiveness of the above improvement measures. The Focal_SIoU loss function (YOLOv8n-F_S) was added to the initial model (YOLOv8n) in sequence, the GAM attention mechanism was added to the backbone part (YOLOv8n-F_SG), and the small target detection layer was added to the neck part (YOLOv8n-F_SGX) to conduct ablation experiments on the cucumber seed germination detection model YOLOv8-F_SGX. For ease of distinction, we have named the improved model YOLOv8n-F_SGX, where F_S represents the introduced Focal_SIoU loss function, G represents the addition of the GAM attention mechanism to the base model, and X represents the added small-object detection layer. The parameters of each model recorded are shown in **Table 5**. Analysis of various data in the table showed that the average precision of the model after adding the Focal_SIoU loss function increased by 1.5% compared with that of the initial model, whereas the Params and FLOPs remained almost unchanged. On this basis, the GAM attention mechanism was continuously added, which increased the mAP by 0.2%. However, due to the introduction of additional neural network layers, the Params and FLOPs of the model increased by 54.7% and 16%, respectively. After the small target detection layer was added, the mAP of the model increased by 1.4%, the Params decreased by 0.6%, and the FLOPs increased by 46.8%. This finding may be attributed to the addition of additional convolutional layers and detection layers and the need for specially designed anchor sizes and ratios to better capture the characteristics of small targets, resulting in an increase in the amount of computation. After the above three improvement measures, the final model demonstrated a significant improvement in accuracy compared with the initial model (an increase of 3.1%). It can also maintain a relatively low model complexity.

**Table 5:** Main indicators of the improved model.

Model	mAP@0.5	Params(M)	FLOPs(G)	Time(h)
YOLOv8n	0.947	3	8.1	0.127
YOLOv8n-F_S	0.962	3	8.1	0.127
YOLOv8n-F_SG	0.964	4.64	9.4	0.132
YOLOv8n-F_SGX	0.978	4.61	13.8	0.199

**Figure 6:**
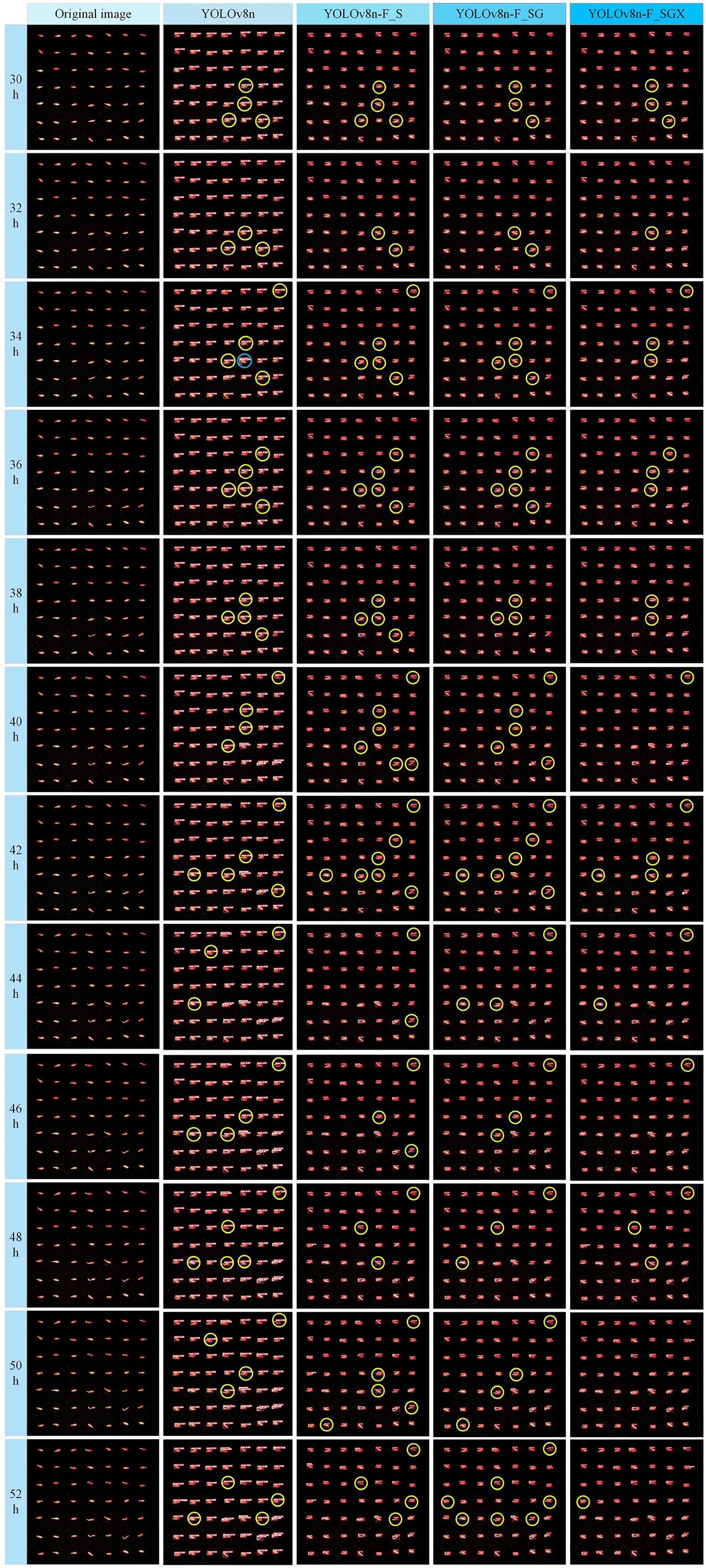
Detection effect diagram of the improved model.

The germination images in the 3–52 h time period, where the initial model had more missed detections, were selected for prediction to compare the detection capabilities of each model for cucumber seeds and radicles more intuitively and verify the improvement effect of the model on the detection of radicles in the early stage of germination. The comparison results are shown in **Figure 6**. After the Focal_SIoU loss function and the GAM attention mechanism were added, no more false detections occurred, but the situation of missed detections did not significantly improve, indicating that the model’s ability to capture and recognize small radicles was still not strong. After the small target detection layer was introduced, the number of missed detections of small radicles in each germination period was significantly reduced. In the detection of germination images at the 40th and 50th h, no missed detections were observed, indicating that the YOLOv8n-F_SGX, after adding the small target detection layer, had a great improvement in the detection ability of small radicle targets. The overall performance of the model basically met the detection requirements for cucumber seeds and radicles.

### 2.7 Selection and evaluation metrics of the seedling stage image segmentation model

YOLOv8-seg is an instance segmentation model in the YOLOv8 series. Based on the instance segmentation solution of YOLACT (You Only Look At CoefficienTs), it can perform effective instance segmentation to achieve the recognition and segmentation of each independent instance in the image (Hu et al., 2024). The main hyperparameters for training the instance segmentation model are the same as those in **Table 3**.

Two indicators, the number of parameters (Params) and the number of floating-point operations (FLOPs), were used to evaluate the complexity of the instance segmentation model. Box (mAP50) and Mask (mAP50) were used to evaluate the detection and segmentation accuracy of the model, respectively. Mean average precision (mAP) is an important performance evaluation metric. As an instance segmentation model, what distinguishes YOLOv8-seg from the object detection model is that Mask (mAP50) is the mean average precision of instance segmentation, calculated at a mask Intersection-over-Union (IoU) threshold of 0.5. It represents the model’s ability to correctly detect and segment individual instances. It represents the degree of matching between the segmentation results predicted by the model and real annotations, and it can help evaluate the positioning and segmentation capabilities of the model. Mask (mAP50) can be calculated by the following Equations 1–5:

(1) $$Mask(mAP_{50})=\frac{1}{N_{cls}}\sum_{c=1}^{N_{cls}}AP^{mask-IoU=0.5}$$

(2) $$Precision=\frac{TP_{0.5}}{TP_{0.5}+FP_{0.5}}$$

(3) $$Recall=\frac{TP_{0.5}}{TP_{0.5}+FN_{0.5}}$$

(4) $$AP_{C}=\int_{0}^{1}P_{c}(R_{C})dR_{C}$$

(5) $$mAP=\frac{1}{C}\sum_{c=1}^{c}AP_{C}$$

where TP_0.5_ is the Number of predicted instances (or bounding boxes) with mask IoU ≥ 0.5 and correct class, FP_0.5_ is the Number of predicted instances with mask IoU < 0.5 or incorrect class, and FN_0.5_ is the number of ground-truth instances that were not detected. Ncls represents the total number of classes; APc represents the average precision (AP) for class c, calculated as the area under the precision-recall curve (PR curve); IoU = 0.5 means that a detection is considered valid only when the Intersection over Union (IoU) between the predicted bounding box and the ground-truth bounding box is ≥0.5. Equation 2 and Equation 3 are used to calculate precision and recall, respectively. TP represents predicted bounding boxes with IoU ≥ 0.5 and correct class; FP represents predicted bounding boxes with IoU < 0.5 or incorrect class; FN represents ground-truth bounding boxes that were not detected; The AP for all classes is calculated using Equation 4, and the average is taken. Finally, mAP50 is calculated using Equation 5.

**Table 6** shows that the Mask (mAP50) of YOLOv8-seg reached 0.987, indicating that this model can achieve a high-precision division of the leaf contours and help accurately quantify the cotyledon area of cucumber seedlings in different time periods.

**Table 6:** Indicator parameters of YOLOv8-seg.

Index	Parameter
Mask P	0.983
Mask R	0.977
Mask(mAP50)	0.987
Params	325M
FLOPs	12.0G

**Figure 7:**
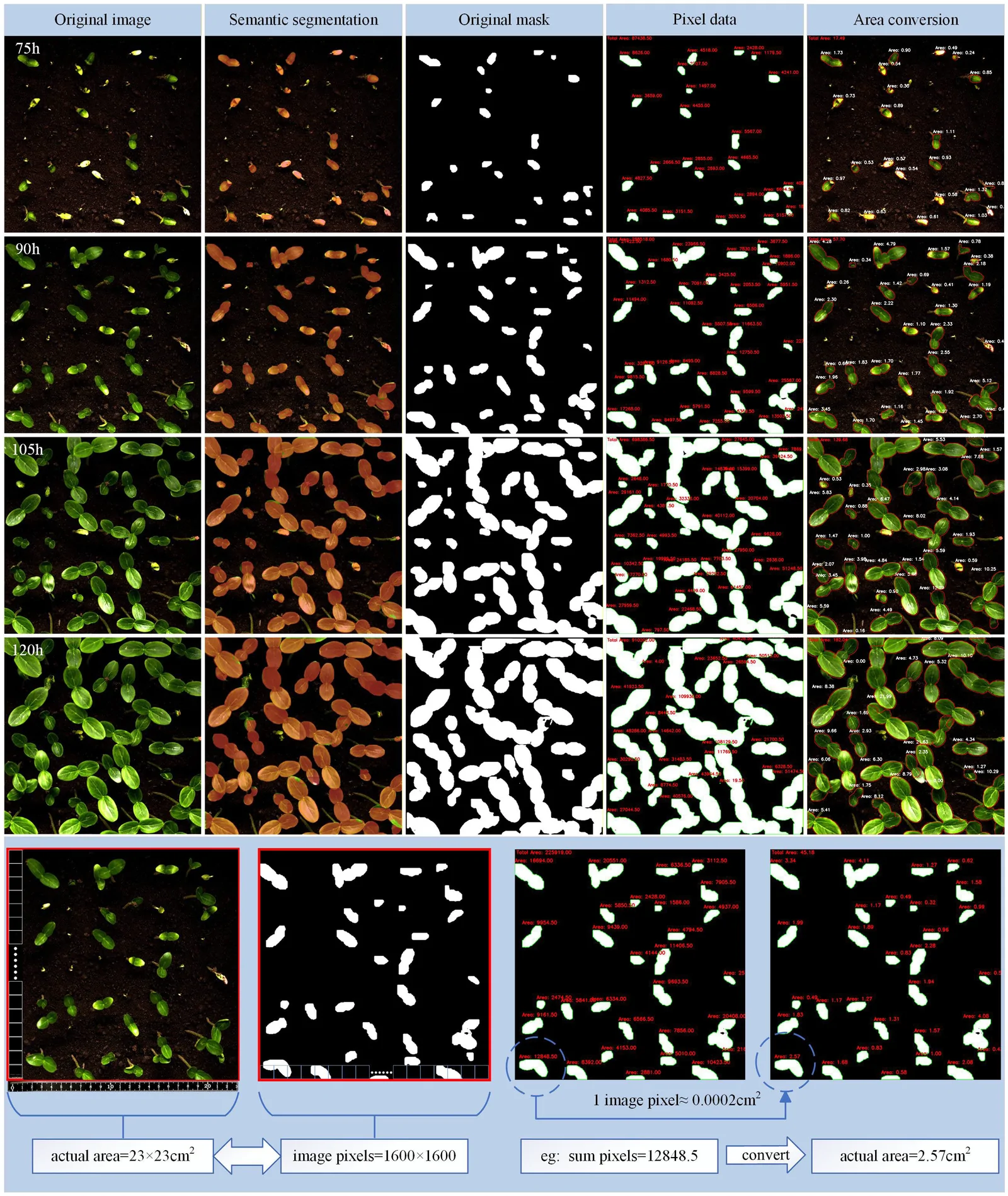
Extraction process of the cotyledon area of cucumber seedlings.

The automatic extraction of the cotyledon area was realized on the basis of the screened segmentation model to evaluate the growth vitality of cucumber seedlings during the seedling stage and explore the degree of vitality correlation between the budding and seedling stages. The specific process is shown in **Figure 7**. First, the original image was inputted, and the abovementioned model was used to perform instance segmentation on the leaf area to extract the mask map (mask) of the leaf. Through the mask map, the contour and shape of each leaf predicted by the model can be clearly seen. The total number of pixels contained in one picture was calculated to be 2,560,000. In accordance with the scale attached to the edge of the soil-rooting box, the actual area of each picture was calculated to be 52900 mm². Then, the conversion scale from the number of pixels to the actual area can be obtained, that is, one pixel approximately corresponds to an actual area of 0.02 mm². Therefore, the real area of each leaf can be calculated through the number of pixels in the mask map of each leaf. Finally, the growth status of the seedlings in different experimental groups can be intuitively compared by superimposing the extracted leaf contours and the leaf area values on the original image.

The detection of cucumber seed germination rates and cotyledon area using this platform can be divided into five steps: crop cultivation, image acquisition, dataset annotation, model training, and vitality metric extraction. of which crop cultivation, image acquisition, and the extraction of vitality indicators are fully automated. The platform enables automated control of the internal crop growth environment, and the trained detection and segmentation model can directly extract vitality indicators such as germination rate and cotyledon area; however, the annotation and creation of image datasets, as well as model training and refinement, still require manual intervention.

**Figure 8:**
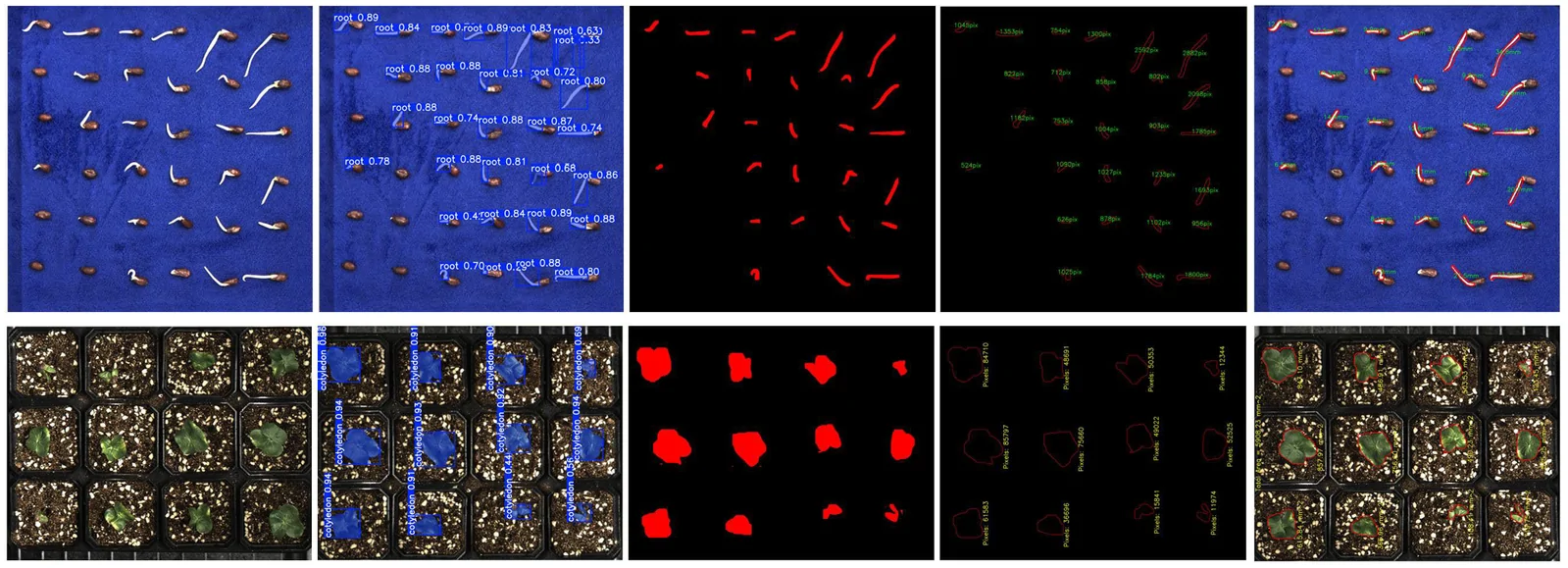
Test results for cotton radicles and cotyledons.

To verify the preliminary generalizability of the vitality monitoring platform developed in this paper, we also collected images of cotton seed growth in addition to those of cucumbers. Using the improved model, we accurately extracted the length of cotton radicles under hydroponic conditions and the area of cotton cotyledons under soil-based conditions. The results are shown in the **Figure 8** below, which clearly demonstrates that the vitality monitoring platform developed in this paper is not only applicable to cucumbers but can also be extended to monitor the growth vitality of other crops.

**Figure 9:**
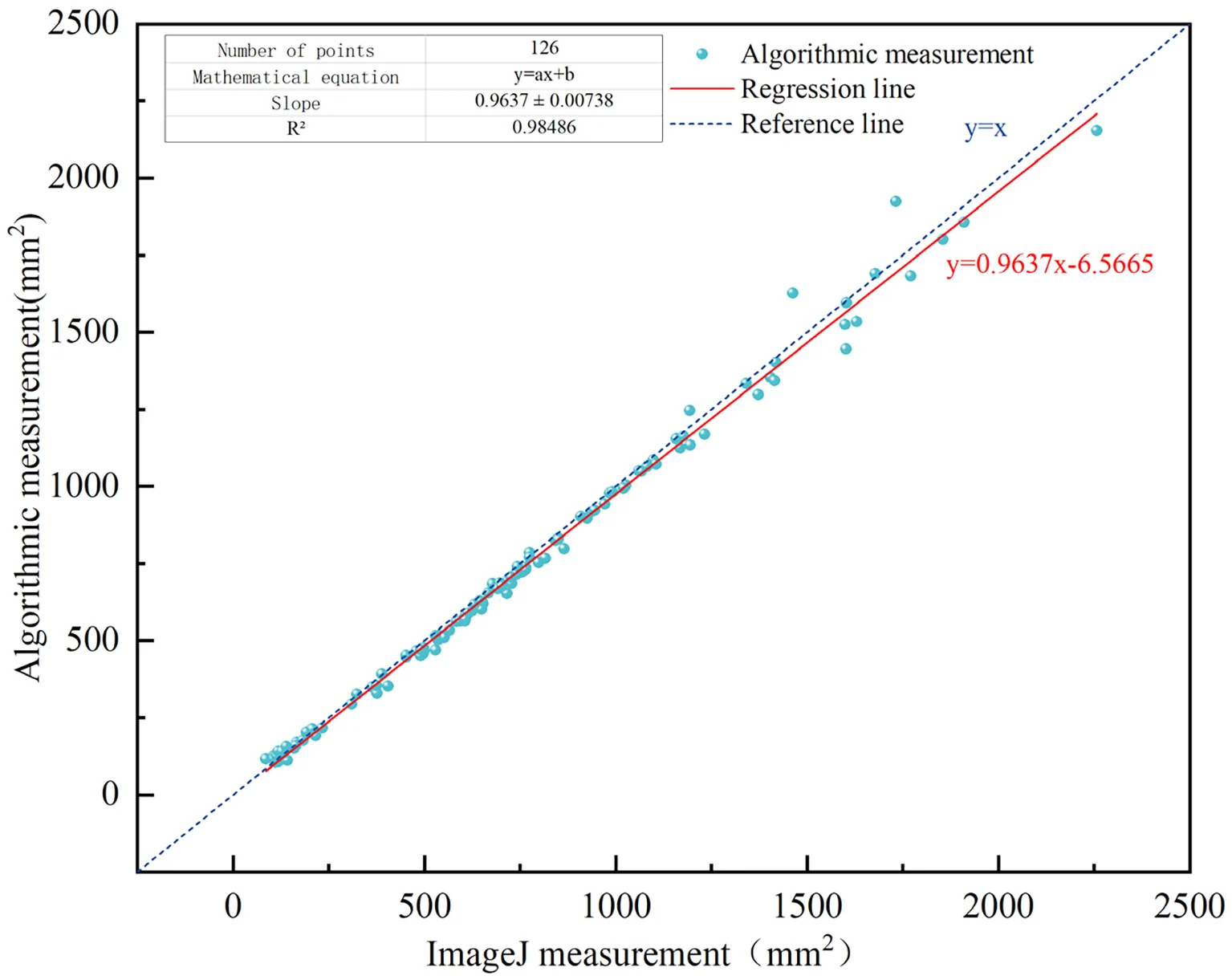
Linear fitting analysis of cucumber cotyledon area at 124h.

To verify the accuracy of the cotyledon area measurement method proposed in this study, this paper used the open-source image processing software ImageJ to manually annotate each cotyledon in the images. A pixel-to-physical unit conversion factor was established based on the scale, and pixel distances and areas were converted to actual physical units. To ensure the randomness of the measurement results, this study randomly selected 126 cotyledons from the acquired images as samples. The algorithm model was used to measure each cucumber cotyledon, and the measurement results were subjected to linear fitting analysis against the results obtained manually using ImageJ, as shown in **Figure 9**.

### 2.8 Experimental design

NaCl solutions with concentrations of 0, 30, 60, 90, 120, and 150 mmol·L^−1^ were prepared using analytically pure NaCl particles(Sinopharm Chemical Reagent Co., Ltd.) and deionized water to simulate different levels of salt stress conditions to investigate the impact of ZnO-NPs priming treatment on the germination and growth vitality of soil-cultivated cucumber seeds under salt stress. A nano-zinc oxide dispersion (Jiangsu Tianxing New Materials Co., Ltd.) with a particle size of 30 nm and a concentration of 30% was used, and five different concentrations (100, 200, 400, 800, and 1600 mg·L^−1^) of ZnO-NPs suspensions were prepared by diluting with deionized water. The cucumber seeds were soaked and shaken in these suspensions, with a total of 36 groups of experiments as shown in **Table 7**. The seeds and different concentrations of ZnO-NPs suspensions were placed in conical flasks, sealed, and shaken on a shaker at 120 r/min. After 6 h, the seeds were taken out and the residual ZnO-NPs suspensions on the seed surface were washed off with deionized water. After the seeds were dried, they were sown in a 7 × 7 arrangement on the soil layer of the soil-rooting box. The soil-rooting box was then placed in an incubator with a constant temperature of 25 °C and a relative air humidity of 70%–90% for the full-time sequence image acquisition of cucumber seed germination and growth, which lasted for 7 days. Each composite experiment was repeated three times, with each seed tray (containing 49 seeds) serving as an independent experimental unit for statistical analysis.

**Table 7:** Composite experimental groups of priming treatments with different concentrations of NaCl solutions and ZnO-NPs suspensions.

Concentration of NaCl(mmol·L^-1^)	Concentration of ZnO-NPs(mg·L^-1^)					
	0(ck)	100	200	400	800	1600
0(ck)	CK	Z100	Z200	Z400	Z800	Z1600
30	N30	N30Z100	N30Z200	N30Z400	N30Z800	N30Z1600
60	N60	N60Z100	N60Z200	N60Z400	N60Z800	N60Z1600
90	N90	N90Z100	N90Z200	N90Z400	N90Z800	N90Z1600
120	N120	N120Z100	N120Z200	N120Z400	N120Z800	N120Z1600
150	N150	N150Z100	N150Z200	N150Z400	N150Z800	N150Z1600

## 3 Results and discussion

### 3.1 Image acquisition results of cucumber seed germination and cotyledon growth

**Figure 10:**
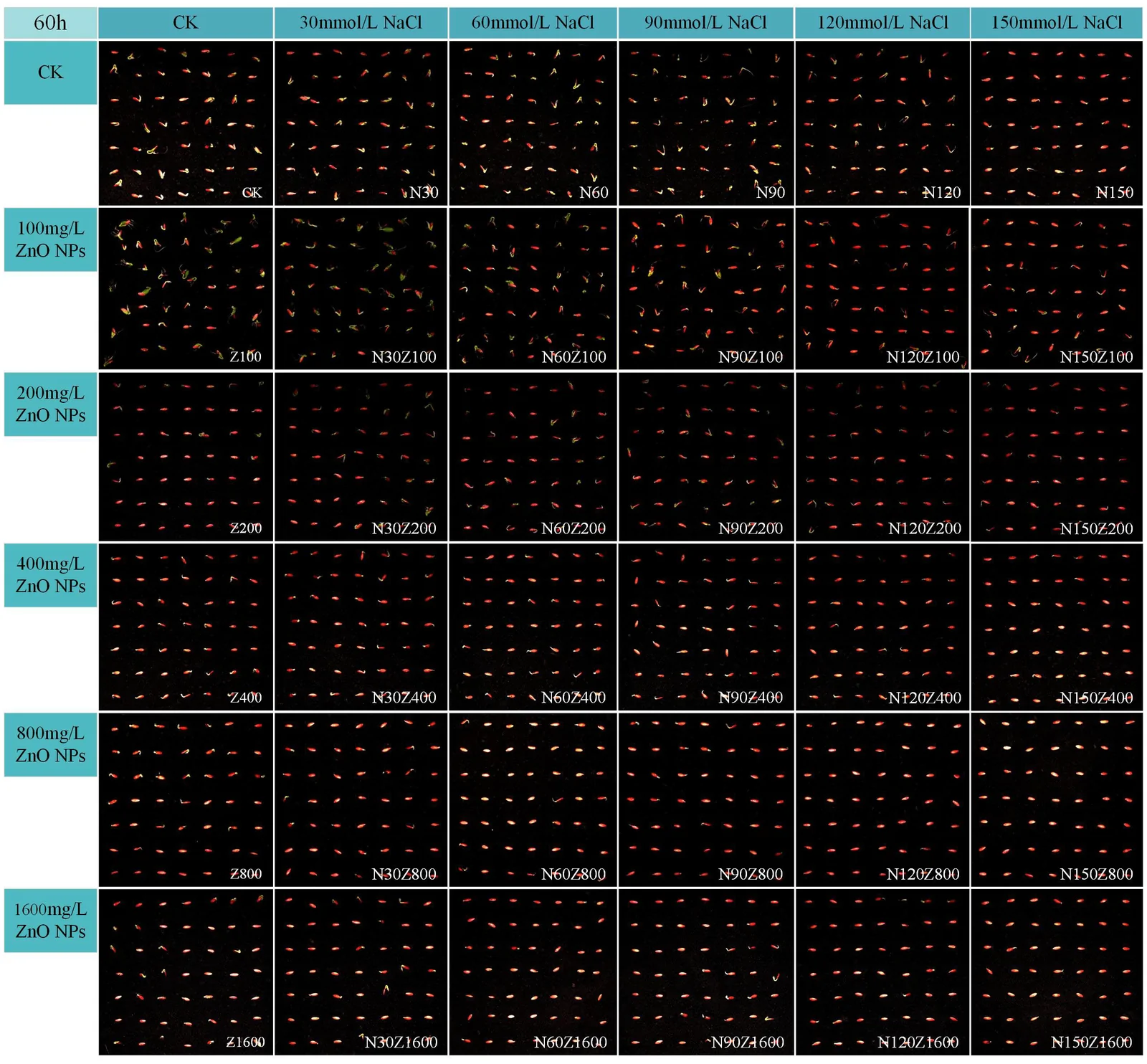
Germination images of cucumber seeds at 60th h under different experimental groups.

**Figure 11:**
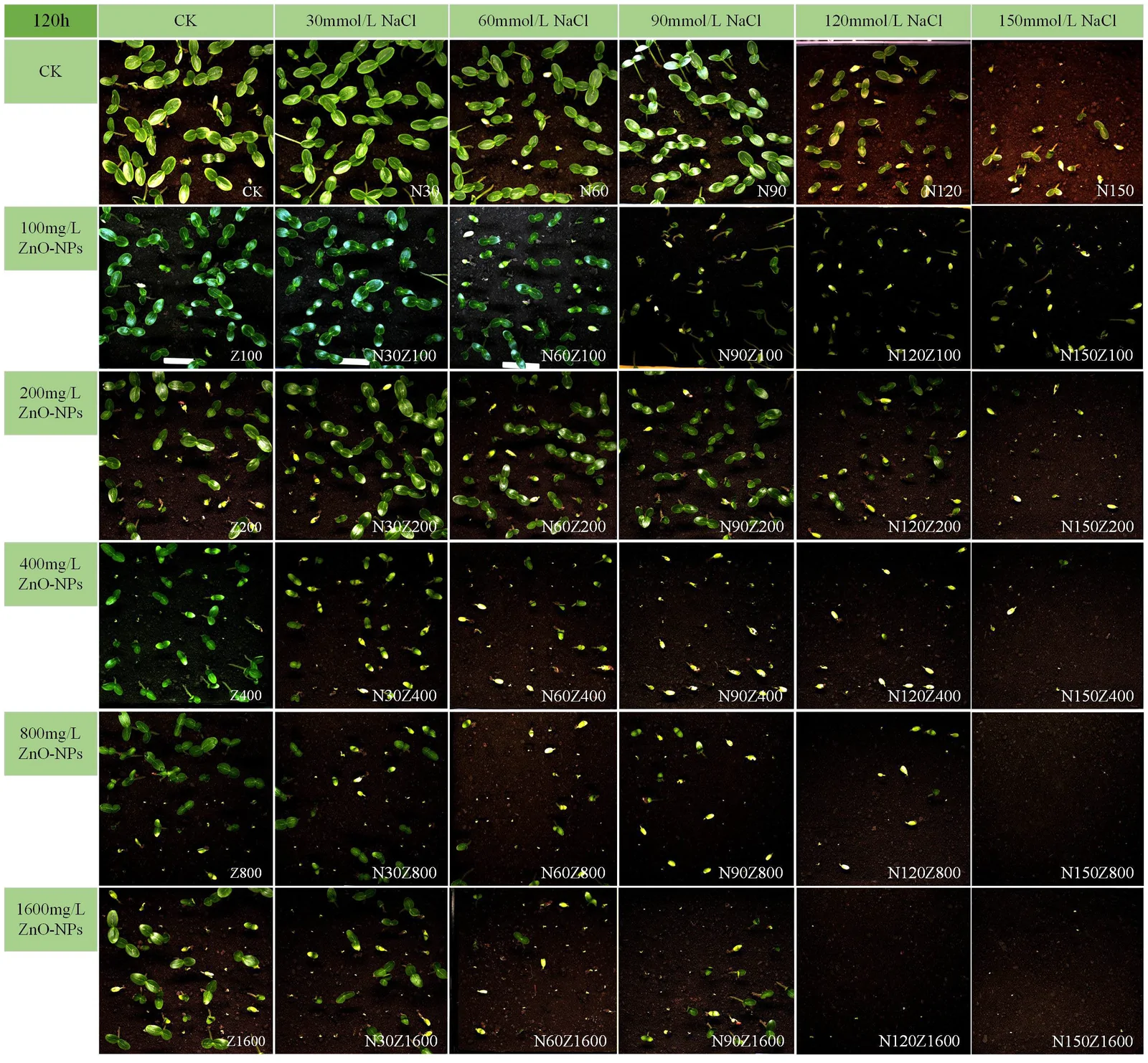
Growth images of cucumber seedlings at 124th h under different experimental groups.

To quantify the growth vigor indicators of cucumber seeds across different experimental groups during the germination and seedling stages, this study utilized the aforementioned full-time-series crop growth monitoring system to capture images at 60 hours post-germination and 120 hours into the seedling stage. In this formal experiment, we treated each tray as an experimental unit, representing a set of composite experiments, with each composite experiment repeated three times. Each tray was populated with 49 seeds that had been treated with ZnO-NP solutions of varying concentrations, and the seeds were cultured while being simultaneously treated with NaCl solutions of varying concentrations. Comparative analysis of the growth imagery revealed the regulatory effects of ZnO-NPs suspension priming on cucumber development under varying concentrations of NaCl stress, as illustrated in **Figure 10** and **Figure 11**.

### 3.2 Indicators of germination during the budding stage and growth vitality during the seedling stage of cucumber seeds

#### 3.2.1 Evaluation indicators of germination vitality during the budding stage

The number of detected radicles was taken to represent the number of germinated seeds, and the following Equations 6–8 were used to calculate the germination rate, germination energy, and germination index to accurately evaluate the germination vitality of cucumber seeds under different experimental treatment groups:

(6) $$Germination\;rate(\%)=(\frac{N_{t}}{N})\times 100\%$$

(7) $$Germination\;energy(\%)=(\frac{N_{48}}{N})\times 100\%$$

(8) $$Germination\;index=\sum (G_{t}/D_{t})$$

where Nt represents the number of germinated seeds at the t-th h, N represents the total number of cultured seeds, N48 is the number of germinated seeds cultured until the 48th h, Gt is the number of germinated seeds within t h, and D t is the corresponding culturing time.

#### 3.2.2 Evaluation indicators of growth vitality during the seedling stage

On the basis of the abovementioned YOLOv8-seg instance segmentation model, the cotyledon area of cucumber seedlings in different time periods can be extracted from the full-time sequence germination and growth images. Therefore, total cotyledon area was chosen as the evaluation indicator of growth vitality. It refers the photosynthetic capacity of seedlings and can serve as an indicator of crop health status and growth rate (Chen et al., 2023), indirectly reflecting the growth vitality of crops and helping us compare the impacts of different concentrations of ZnO-NPs suspension priming treatments on the growth vitality of cucumber seedlings under salt stress. Considering that the area of each culturing box was the same, the total cotyledon area of the seedlings in each image was counted for the convenience of comparison.

#### 3.2.3 Impact of soaking cucumber seeds with ZnO-NPs on the germination of cucumber seeds and the growth of seedlings under salt stress

**Figure 12:**
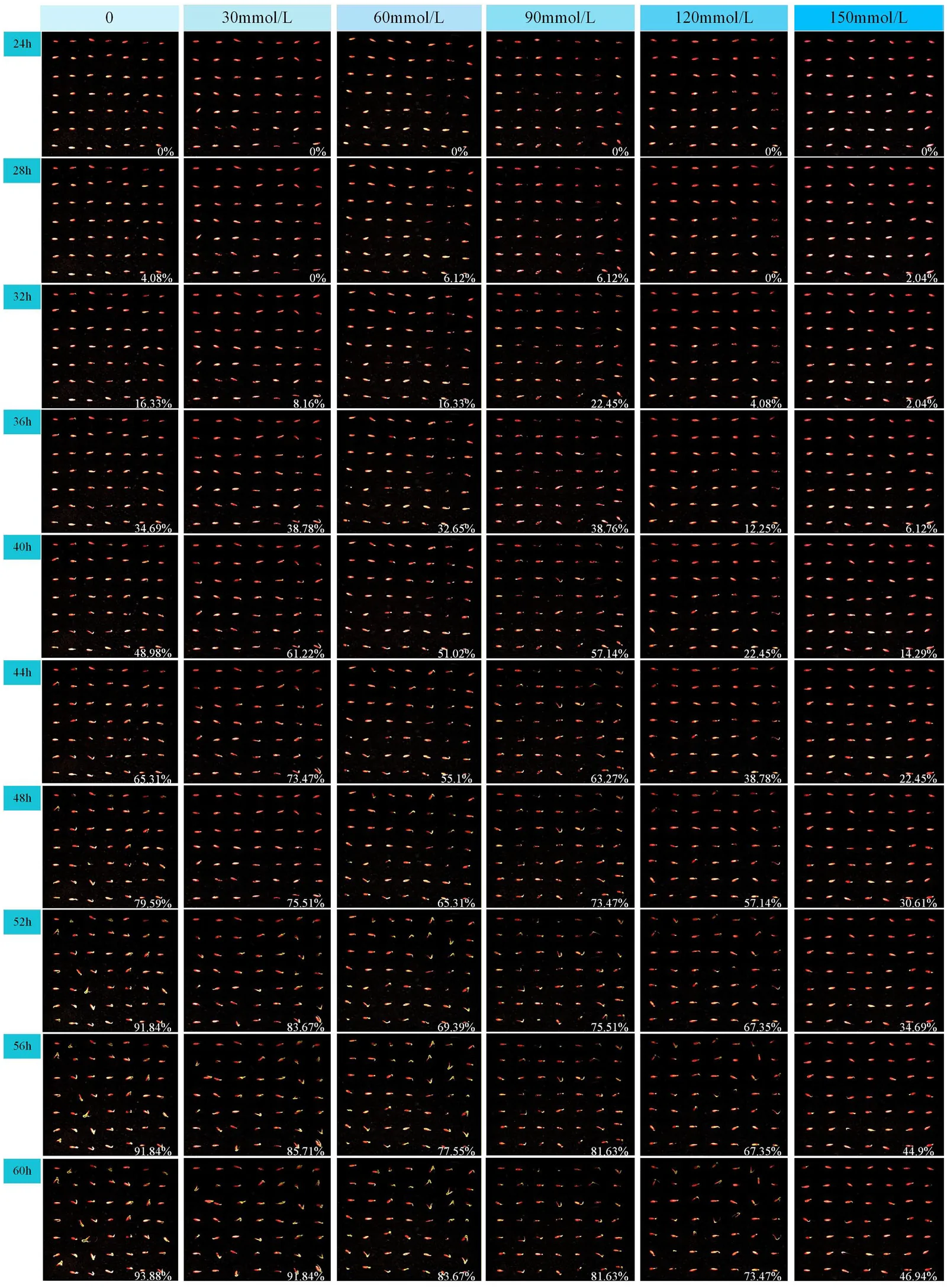
Germination images of cucumber seeds cultivated in NaCl solutions with different concentrations.

**Figure 13:**
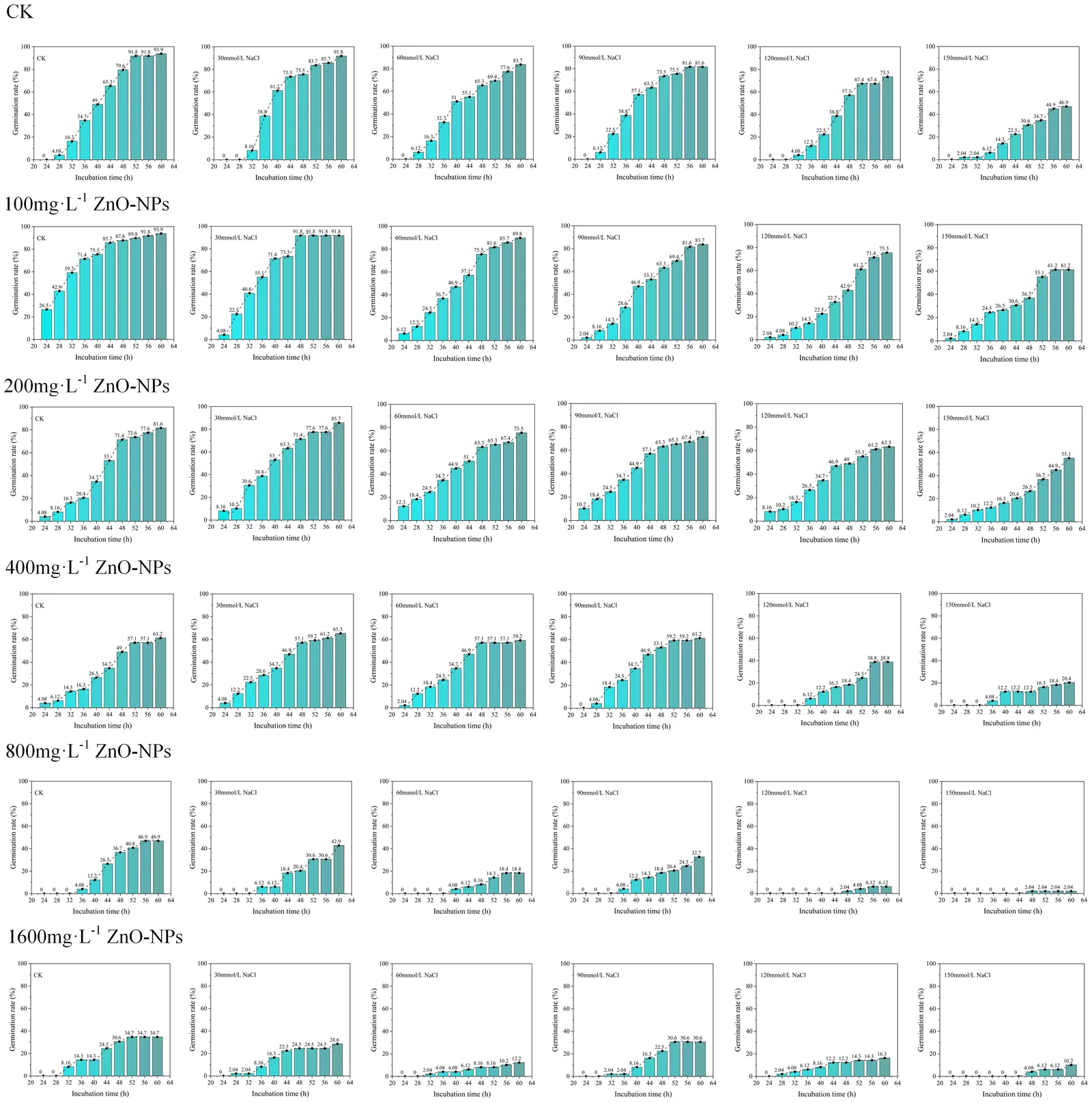
Statistical chart of the germination rate of cucumber seeds under different experimental groups.

The germination images of 60 h from 36 experimental groups were obtained using the crop cultivation monitoring platform to explore the mitigation effects of different concentrations of ZnO-NPs on salt stress. The germination images of different experimental groups were detected and recognized through the above-improved YOLOv8n-F_SGX object detection model. The numbers of “seed” and “root” in different stages of each experimental group were counted. **Figure 12** shows the germination rates of the control group at different time points. The changing trends of the germination rate are shown in **Figure 13**. Although the cotyledon area under 100 mg·L^−1^ treatment was only slightly higher than that of the corresponding controls, the consistent acceleration in germination rate and germination index across all salt stress levels suggests an overall promoting trend within the tested concentration range.

The germination test is a basic method for determining the impact of nanoparticles on plant growth. Germination images were selected to study the influence of ZnO-NPs suspensions on the germination vitality of cucumber seeds under different levels of salt stress, and the corresponding germination rates every 4 h from the 24th h to the 60th h were counted. **Figure 11** shows the changing trends of the germination rates of cucumber seeds in the control group and those treated with different concentrations of ZnO-NPs suspensions under six salt stress environments.

When the cucumber seeds were treated with different concentrations of ZnO-NPs suspensions, the cucumber seeds in the control group generally started to germinate after 28 h or even 32 h. However, after being treated with ZnO-NPs suspensions at the concentrations of 100 and 200 mg·L^−1^, the seeds began to germinate at 24 h, which was about 4–8 h earlier than the control group, and the germination rate in the early stage was higher than that of the control group, with the germination process significantly accelerated.

**Figure 14:**
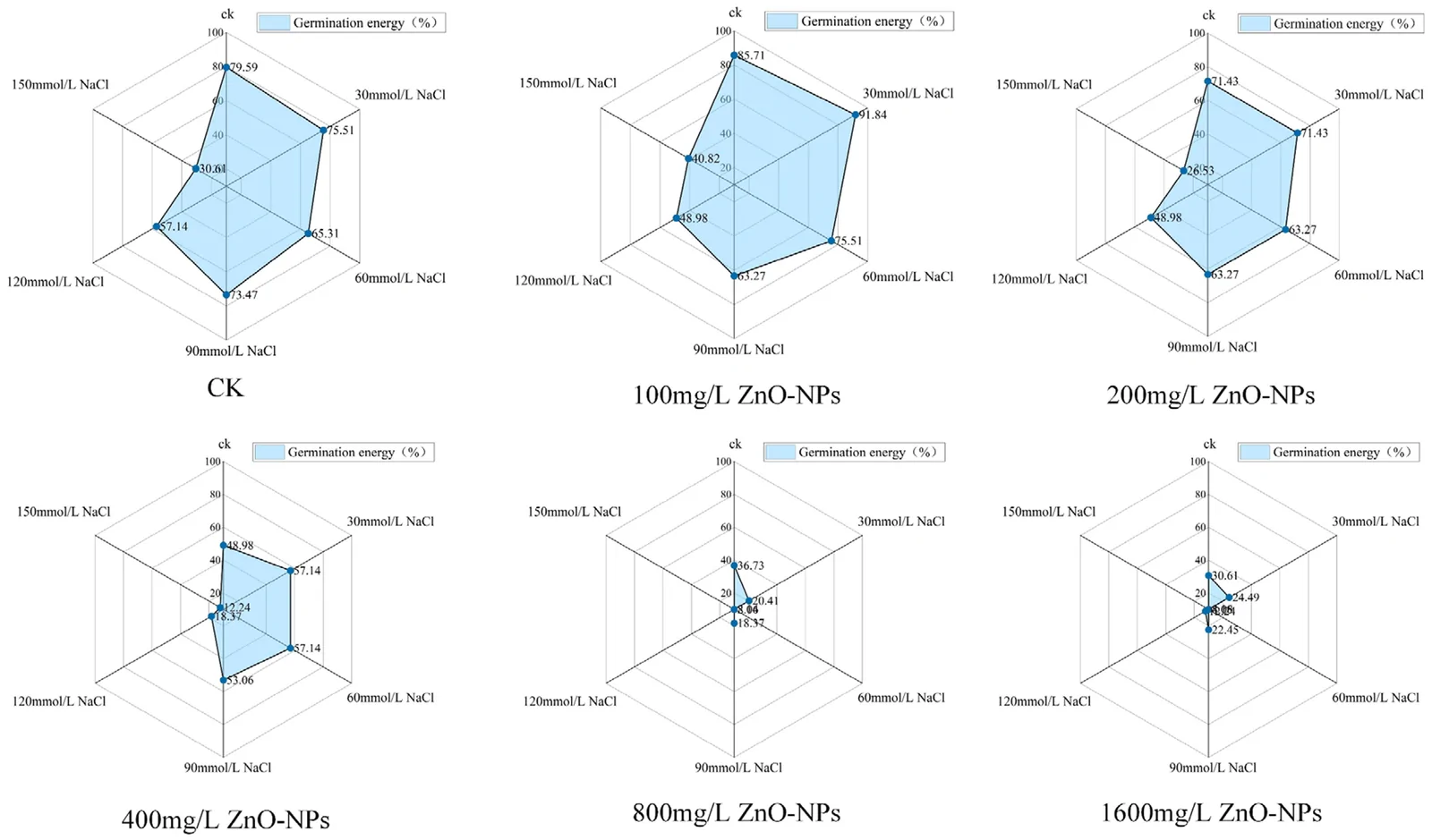
Statistical chart of the germination energy of cucumber seeds under different experimental groups.

**Figure 15:**
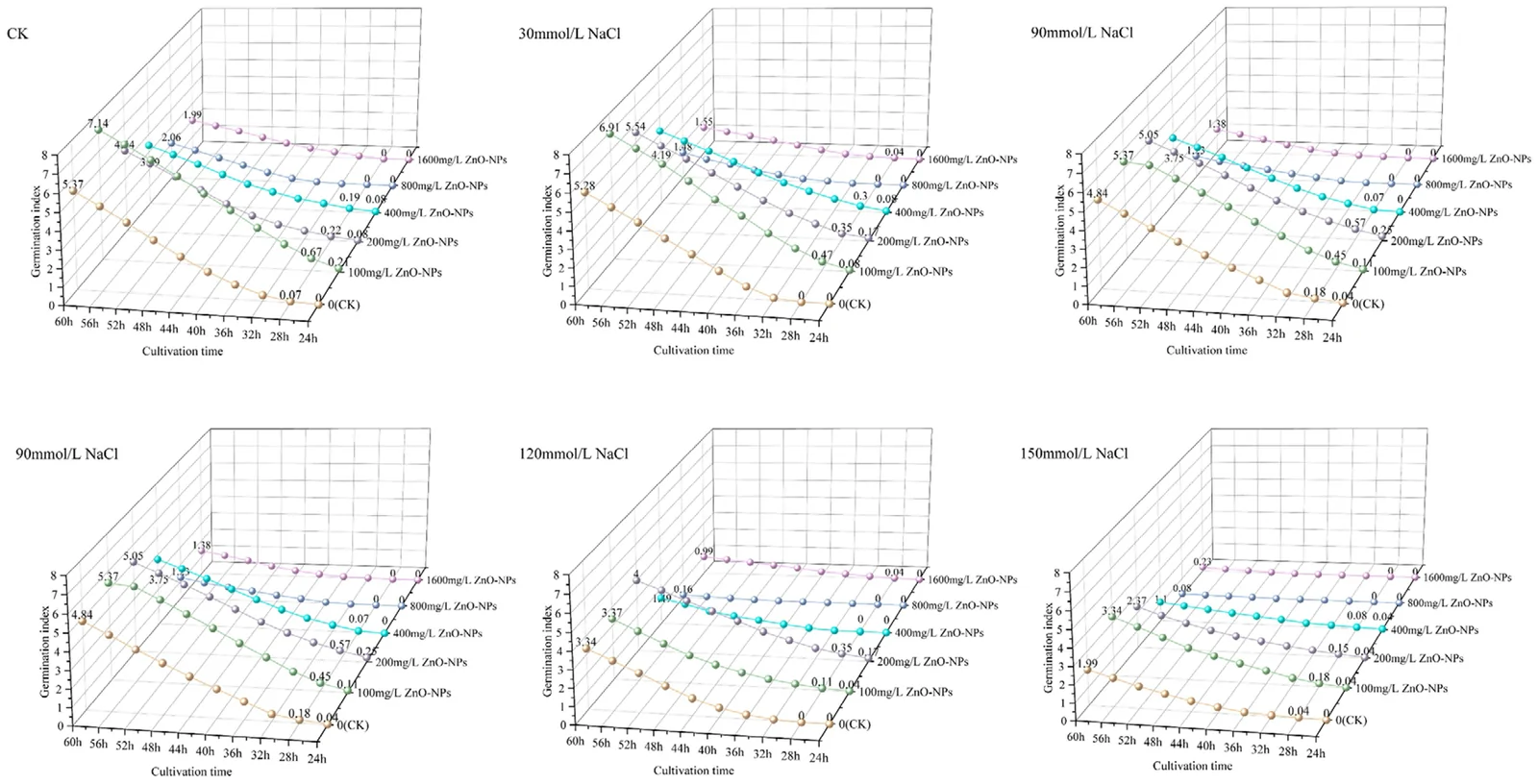
Statistical chart of the germination index of cucumber seeds under different experimental groups.

As indicators of seed vitality, the germination energy and the germination index can reflect the speed and vitality of seed germination. The higher the values of these two indicators, the stronger the vitality of the seeds. **Figures 14**, **15** show that the germination indices of the treatment groups at the concentrations of 100 and 200 mg·L^−1^ in the early stage under different levels of salt stress were all higher than those of the control group CK.

However, when the concentration of the ZnO-NPs suspension was 400 mg·L^−1^ or above, the germination rates and indices of the cucumber seeds under different levels of salt stress within the same time period decreased significantly, indicating that an excessively high concentration of the ZnO-NPs suspension could be toxic to the cucumber seeds and inhibit their germination.

Based on the comprehensive analysis of the above experimental data, the treatment with a low-concentration ZnO-NPs suspension can accelerate the early germination of cucumber seeds and relieve the inhibitory effect of salt stress on the early germination vitality of cucumber seeds, whereas the treatment with a high-concentration ZnO-NPs suspension inhibited the germination of cucumber seeds throughout the whole period, and the higher the concentration, the stronger the inhibitory effect.

**Figure 16:**
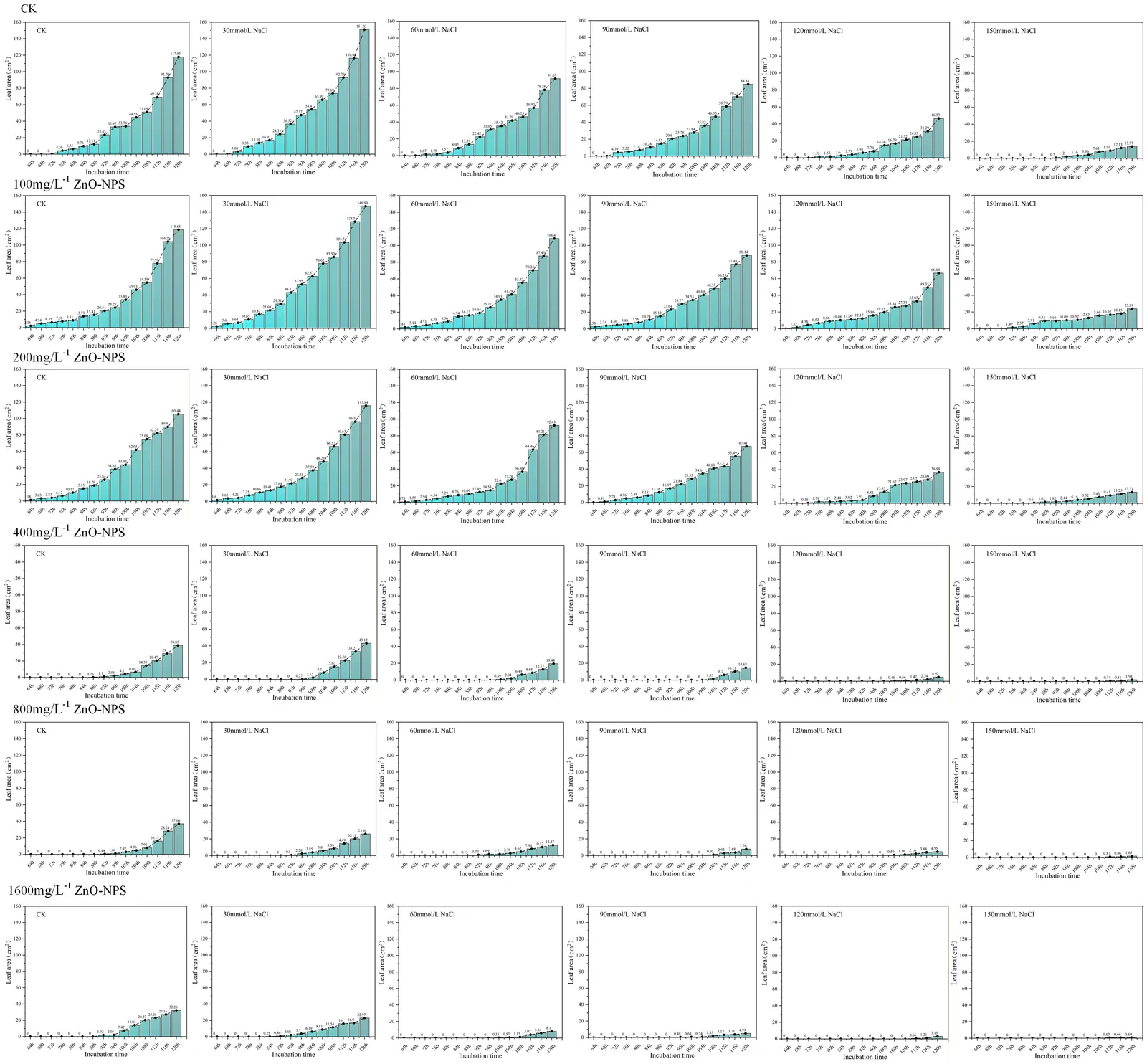
Statistical chart of the total cotyledon area of cucumber seedlings under different experimental groups.

From 64 h to 120 h, growth images of 36 experimental groups were selected every 4 h, and the corresponding total cotyledon area was calculated to investigate whether the ZnO-NPs induction treatment could continue to affect the growth of cucumber seedlings, as shown in **Figure 16**. Longitudinal comparison of the six groups of experiments showed that under the priming treatment of the ZnO-NPs suspension at a concentration of 100 mg·L^−1^, the total cotyledon areas of Z100, N30Z100, N60Z100, N90Z100, N120Z100, and N150Z100 at the 120th h were 11868, 14699, 10840, 8814, 6664, and 2339 mm², respectively, which were slightly higher than those of the control group CK, N30, N60, N90, N120, and N150. The experimental results indicated that the priming of ZnO-NPs suspension at a concentration of 100 mg·L^−1^ has a certain promoting effect on the cotyledon growth of cucumber seedlings under salt stress.

When the concentration of the ZnO-NPs suspension was 400 mg·L^−1^, the total cotyledon areas of Z400, N30Z400, N60Z400, N90Z400, N120Z400, and N150Z400 at the 120th h were 3885, 4312, 1944, 1468, 492, and 198 mm², respectively. When the concentration of the suspension was further increased to 800 and 1600 mg·L^−1^, the total cotyledon areas decreased significantly, indicating that increasing the concentration of ZnO-NPs suspension can exhibit an inhibitory effect on the growth of cotyledons, and the higher the concentration, the stronger the inhibitory effect. The concentration-dependent effects of ZnO-NPs observed in this study are consistent with the hormesis theory, where low-dose nanoparticles may act as mild stress signals that activate antioxidant defenses and promote seed metabolism, while high doses induce oxidative stress, cytotoxicity, and physical barriers to water uptake (Dali et al., 2025; Resente and Crivellaro, 2025; Asif et al., 2025). However, as our experiment focused on phenotypic responses rather than physiological or molecular mechanisms, we refrain from drawing definitive mechanistic conclusions. Further investigations involving physiological indicators (e.g., antioxidant enzyme activity, ROS levels) are needed to elucidate the underlying pathways.

## 4 Conclusion

The following research works were carried out to quickly and accurately determine the early growth vitality of cucumber seeds under soil cultivation conditions and explore the impact of different concentrations of ZnO-NPs suspension priming treatments on the early growth vitality of cucumber seeds under salt stress:

1. A crop cultivation monitoring platform that integrates seed germination cultivation, environmental control, and image acquisition was developed. A soil cultivation germination experiment of cucumber seeds was conducted, full-time sequence germination and growth images were collected within 60 h of the budding stage and 60 h of the seedling stage, and a labeled dataset of the cucumber seed germination and seedling growth processes was constructed on this basis.
2. In accordance with the detection requirements for seeds and radicles, the YOLOv8n-F_SGX object detection model suitable for the detection of soil-cultivated cucumber seeds and radicles was obtained through improvement measures such as adding the Focal_SIoU loss function in addition to the YOLOv8n, adding the GAM attention mechanism to the backbone network, and adding a small target detection layer. These measures enabled the measurement of the germination rate of cucumber seeds. Meanwhile, the division of the cotyledon leaf contours of cucumber seedlings was achieved using the YOLOv8-seg instance segmentation model, and the TOTAL COTYLEDON AREA of the cotyledons of cucumber seedlings was accurately converted in accordance with the number of binary image pixels in the leaf area.
3. Six different concentrations of ZnO-NPs suspensions were used to prime cucumber seeds, and 36 groups of germination experiments were performed under six kinds of salt stress. On the basis of YOLOv8n-F_SGX and YOLOv8-seg, the vitality indicators, such as the germination rate, germination energy, germination index, and cotyledon area index, were quantified, and the germination and growth vitality of cucumber seeds in the 36 groups of experiments were analyzed. The horizontal and vertical comparisons between different experimental groups demonstrated that the priming treatment with low-concentration ZnO-NPs (100 mg·L^−1^) can accelerate the early germination of cucumber seeds and promote the cotyledon growth of seedlings and relieve the inhibitory effect of salt stress on the germination vitality of cucumber seeds in the early stage. Meanwhile, the priming treatment with high-concentration ZnO-NPs can inhibit the germination and cotyledon growth of cucumber seeds throughout the whole period, and the higher the concentration, the stronger the inhibitory effect.

Due to the limited capacity of the incubators in the real-time crop growth vitality monitoring system, the images that can be captured during cucumber seed germination and seedling growth are restricted to top-down views. Consequently, only a limited number of vitality indicators—such as germination rate and cotyledon area—can be extracted. For monocotyledonous crops with fibrous roots and tilted cotyledons, top-down images from a single perspective often fail to provide sufficient biomass information. In subsequent research, the incubator system could be further improved and expanded by adding industrial cameras from other angles to the existing image acquisition module to enable multi-angle image capture. By integrating these multi-angle images, three-dimensional point clouds of seeds and seedlings can be extracted and reconstructed, thereby enabling the extraction of multidimensional biomass information—such as plant height, stem diameter, leaf inclination angle, and seed volume, enabling a comprehensive assessment of crop growth vitality. The preliminary results on cotton suggest that the platform has the potential to be extended to monitor the growth vitality of other crops, although quantitative validation on independent datasets for each crop is still required. Quantitative evaluation on an independent cotton dataset will be conducted in future work to fully validate the platform’s generalization performance.

The crop cultivation monitoring platform developed in this paper and the method for quickly and batchwise determining the vitality of cucumber seeds based on deep learning could provide references for the evaluation system of salt tolerance of other vegetable crop seeds. The experiment on the impact of ZnO-NPs priming treatment on the early growth of cucumbers under salt stress carried out in this paper could provide meaningful references for the subsequent exploration of using nanoparticles to improve the salt tolerance of cucumbers. Although the current platform demonstrated reliable automated image acquisition and vitality metric extraction during the 7-day germination and seedling stage, its long-term stability over extended cultivation periods (e.g., vegetative or reproductive stages) remains to be systematically evaluated (Zhu et al., 2024). Future work will focus on enhancing the system’s durability—such as improving environmental control components and expanding image acquisition capabilities—to support long-term monitoring of later growth stages.

## Data Availability

The raw data supporting the conclusions of this article will be made available by the authors, without undue reservation.
